# Mobile population dynamics and malaria vulnerability: a modelling study in the China-Myanmar border region of Yunnan Province, China

**DOI:** 10.1186/s40249-018-0423-6

**Published:** 2018-04-29

**Authors:** Tian-Mu Chen, Shao-Sen Zhang, Jun Feng, Zhi-Gui Xia, Chun-Hai Luo, Xu-Can Zeng, Xiang-Rui Guo, Zu-Rui Lin, Hong-Ning Zhou, Shui-Sen Zhou

**Affiliations:** 1 Department of Malaria, National Institute of Parasitic Diseases, Chinese Center for Disease Control and Prevention, 207 Rui Jin Er Road, Shanghai, 200025 People’s Republic of China; 2 WHO Collaborating Centre for Tropical Diseases, 207 Rui Jin Er Road, Shanghai, 200025 People’s Republic of China; 3 National Center for International Research on Tropical Diseases, Ministry of Science and Technology, 207 Rui Jin Er Road, Shanghai, 200025 People’s Republic of China; 4 Key Laboratory of Parasite and Vector Biology, Ministry of Health, 207 Rui Jin Er Road, Shanghai, 200025 People’s Republic of China; 5 Yunnan Institute of Parasitic Diseases, Puer, People’s Republic of China; 6 Yingjiang County Center for Disease Control and Prevention, Dehong, People’s Republic of China

**Keywords:** Malaria, Importation, Vulnerability, Mobile population, Individual-based model

## Abstract

**Background:**

The China-Myanmar border region presents a great challenge in malaria elimination in China, and it is essential to understand the relationship between malaria vulnerability and population mobility in this region.

**Methods:**

A community-based, cross-sectional survey was performed in five villages of Yingjiang county during September 2016. Finger-prick blood samples were obtained to identify asymptomatic infections, and imported cases were identified in each village (between January 2013 and September 2016). A stochastic simulation model (SSM) was used to test the relationship between population mobility and malaria vulnerability, according to the mechanisms of malaria importation.

**Results:**

Thirty-two imported cases were identified in the five villages, with a 4-year average of 1 case/year (range: 0–5 cases/year). No parasites were detected in the 353 blood samples from 2016. The median density of malaria vulnerability was 0.012 (range: 0.000–0.033). The average proportion of mobile members of the study population was 32.56% (range: 28.38–71.95%). Most mobile individuals lived indoors at night with mosquito protection. The SSM model fit the investigated data (*χ*^2^ = 0.487, *P* = 0.485). The average probability of infection in the members of the population that moved to Myanmar was 0.011 (range: 0.0048–0.1585). The values for simulated vulnerability increased with greater population mobility in each village.

**Conclusions:**

A high proportion of population mobility was associated with greater malaria vulnerability in the China-Myanmar border region. Mobile population-specific measures should be used to decrease the risk of malaria re-establishment in China.

**Electronic supplementary material:**

The online version of this article (10.1186/s40249-018-0423-6) contains supplementary material, which is available to authorized users.

## Background

Globalization and international population migration have caused imported malaria cases to become the predominant threat to the Chinese malaria elimination program [1, 2]. One major challenge is cross-border malaria transmission, which is a particular concern in the China-Myanmar border region [3–5]. Yingjiang is a county in the Yunnan Province, located at the China-Myanmar border; this region had the majority of national indigenous malaria cases reported in previous years. Therefore, this is a critical region to assess the risk of malaria re-establishment.

In addition to receptivity, malaria vulnerability is considered a major characteristic for risk assessment of malaria re-establishment [6–8]. According to the World Health Organization (WHO) framework for malaria elimination, malaria vulnerability is defined as either the probability of malaria parasite importation into a country or area, or the frequency of the influx of infected individuals, groups, and/or infective anopheline mosquitoes. However, since it is difficult to quantify the importation of infective mosquitoes, imported cases or asymptomatic infections are generally used to quantify vulnerability [6, 7, 9, 10].

Many researchers have found that vulnerability is related to population mobility [2, 3], and preventing the infection of a mobile population in a malaria-endemic area can effectively reduce the importation rate. Thus, it is important to understand the relationship between malaria vulnerability and characteristics of mobile populations. These characteristics include the proportion of mobile individuals in a population of a given area, exposure risk, and the frequency and duration of population movement. Mathematical models are frequently used to quantify a study population’s characteristics, but these models may not always be based on traditional epidemiological methods. The stochastic individual-based model (IBM) and an ordinary differential equation model are commonly used in the quantification process [11–19]. The IBM is also used to assess the risk of malaria establishment [20, 21], although no studies have used these models to examine population mobility and malaria in the China-Myanmar border region. Therefore, by adapting some key components from the IBM model (i.e., simulation based on individuals using a random function), we developed a stochastic simulation model (SSM) using community-based, cross-sectional data to evaluate population mobility and its effect on malaria vulnerability in Yingjiang county.

## Methods

### Study setting

**Fig. 1:**
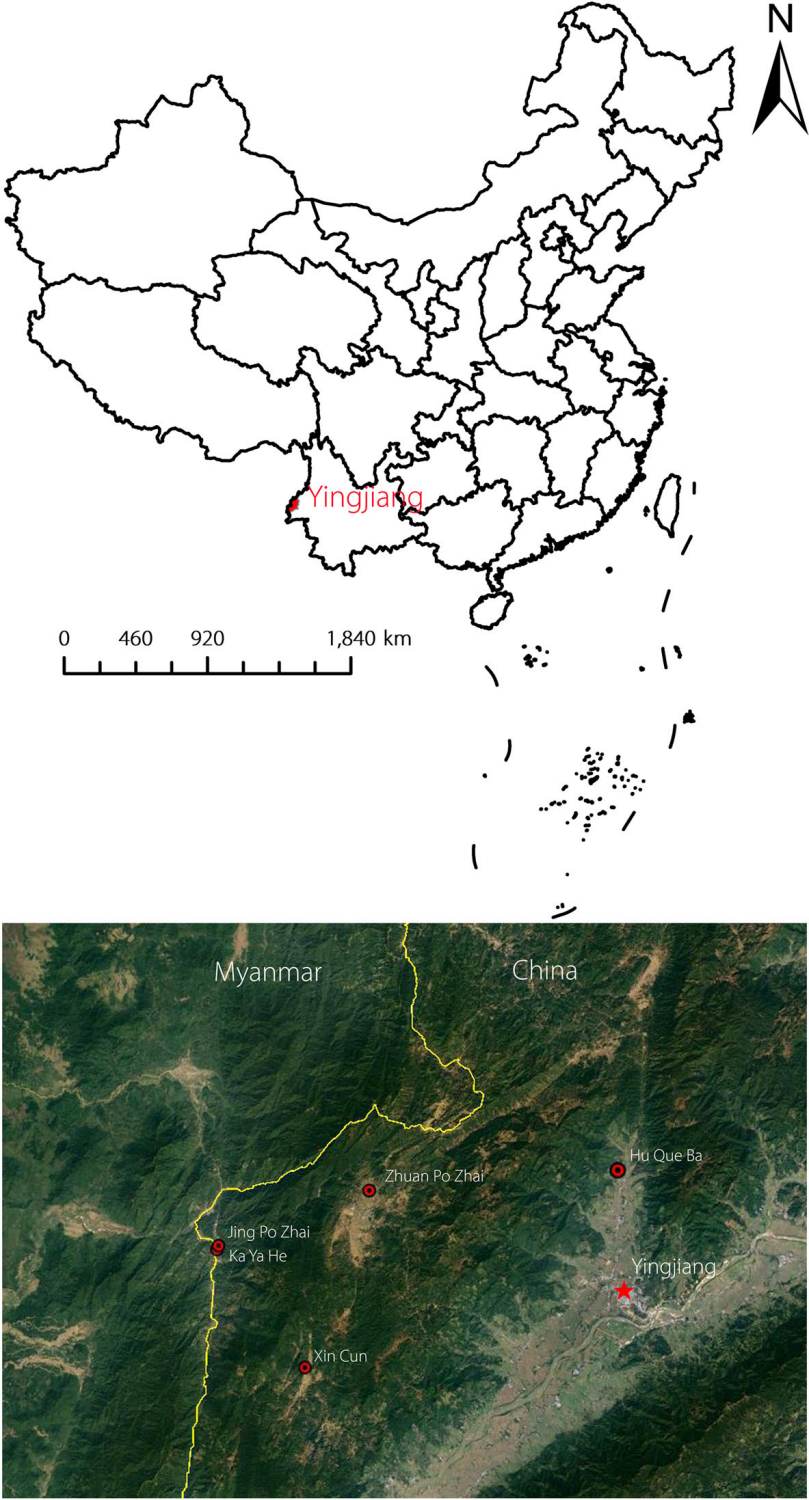
Location of Yingjiang County as well as the five selected villages

A community-based, cross-sectional survey was used to obtain data from five villages (Jing Po Zhai, Ka Ya He, Xin Cun, Zhuan Po Zhai, and Hu Que. Ba) in Yingjiang county of China (western Yunnan Province) (Fig. 1), which is 1 of 18 counties located at the China-Myanmar border. This county shares a 214.6 km border with the Myanmar state of Kachin. The population of Yingjiang county is 307 960 individuals, with cross-border trade, logging, mining, and plantation activities being common. The basic characteristics of the five selected villages are shown in Table 1.

**Table 1:** Epidemiological features of the mobile population and basic information regarding the five selected villages at the China-Myanmar border

	Jing Po Zhai	Ka Ya He	Xin Cun	Zhuan Po Zhai	Hu Que Ba
Terrain	Hilly areas	River valley	Hilly areas	Mountain	Plain
Average temperature (°C)	22	22	16	14	18
Rainfall (mm)	2500	2550	2300	2600	2200
Main crops	Banana	Banana	Rice	Rice	Rice
Number of households	39	22	24	53	32
Number of permanent residents	146	86	82	107	74
Number of mobile population	46	28	59	36	21
Gender (Male/Female)	22/24	13/15	28/31	16/20	9/12
Age (Years)					
0–10	5	3	4	3	2
11–20	5	1	6	3	2
21–30	20	8	14	6	7
31–40	7	7	17	6	4
41–50	2	2	7	8	4
51–60	4	3	10	6	1
> 60	3	4	1	4	1
Immigrant	4	2	0	6	3
Area 1	4	0	0	0	0
Myanmar	4	0	0	0	0
Area 2	0	0	0	0	0
Area 3	0	2	0	6	2
China-Myanmar border areas in China	0	2	0	6	2
Area 4	0	0	0	0	1
Transmission interruption areas in China	0	0	0	0	1
Emigrant	42	26	59	30	18
Area 1	23	8	46	3	8
Myanmar	23	8	46	3	7
The Myanmar-Thailand border region	0	0	0	0	1
Area 2	0	0	0	0	0
Area 3	12	6	6	17	1
China-Myanmar border areas in China	12	6	6	17	1
Area 4	7	12	7	10	9
Transmission interruption areas in China	7	12	7	10	9

### Data collection

The epidemiological survey was performed during September 2016, which is the peak month for local malaria transmission. Before the survey, a pre-survey of four households was conducted in Hu Que. Ba to adjust the previously developed questionnaire and to improve the planning of the survey. There are 170 households located in the five villages, and an area sampling method that included all households was adopted to collect the basic information for each village and its residents. The basic information for each village was collected by interviewing the primary public health provider, and included the village name, terrain, average temperature, rainfall, main crops, number of households, and number of permanent residents (Additional file 1). The information for each individual was collected by interviewing people in each household, and one adult who could provide complete responses for all household members or visitors was interviewed to complete the standardized questionnaire (Additional file 2). Before the survey, the primary public health providers were asked to give the local residents a notice that included the interview date and survey objective, in order to ensure that one adult was at home during the survey. The questionnaire included all family members’ demographic information (age, sex, occupation, education), temporary emigrant information (country, frequency of movement, duration of stay), temporary immigrant information (country, frequency of movement, duration of stay), and categorical exposure risk level (living indoors at night with protection, living indoors at night without protection, living outdoors at night with protection, and living outdoors at night without protection). Protection was defined as the use of a screen door or window, repellent, and/or bed nets, including normal bed nets, long-lasting insecticidal nets, or insecticide-treated nets.

An emigrant was defined as someone who had moved away from the selected village during the previous year. An immigrant was defined as someone who had moved from another place (e.g., Myanmar) into the selected village during the previous year. The mobile population was defined as individuals who had resided in at-risk areas for > 1 night during the previous year, based on the malaria transmission route, although individuals were excluded from the mobile portion of the study population if they performed many daytime border crossings. The proportion of the mobile population was defined as the mobile population divided by the number of permanent residents.

All individuals, including mobile individuals who were residing in the village during the survey, were selected for a serosurvey. Finger-prick blood samples were obtained after each individual provided written informed consent. An 18S rRNA nested polymerase chain reaction (PCR) test and real-time PCR were used to detect *Plasmodium* spp. using the finger-prick blood samples [22, 23].

### Case definition and classification

Data from malaria cases in the studied villages were reported to the web-based National Notifiable Infectious Disease Reporting Information System, with cases between 2013 and 2016 used in the analysis. Data included sex, age, date of illness onset, *Plasmodium* spp., and imported or indigenous case status. Malaria cases were classified as clinically diagnosed or laboratory-confirmed cases, which were both considered eligible for this study. Clinically diagnosed cases were defined as patients with malaria-like symptoms who had lived in or recently travelled to areas with known malaria transmission. Laboratory-confirmed cases were defined as clinically diagnosed cases with positive results from microscopy evaluation for malaria parasites, rapid diagnostic tests, and/or PCR tests [24].

For case classification especially the identification of indigenous or imported cases, there was a step-by-step protocol utilized that was based on dominated specific species, clear seasonality in China, and history of travel. If the case was confirmed as non *P. vivax* in the reference laboratory system, it would be classified as an imported case since only *P. vivax* transmission occurs in China, except around the Yunnan border region. However, classification would be more complicated if the case was diagnosed as a *P. vivax*: based on the individual case investigation, if the onset occurred in the non-transmission season, it would be mostly classified as an imported case without the history of infection or an old infection with the history of infection, while if the onset occurred in the transmission season, it would be carefully classified as an imported case if an individual had a history of travel to malaria-endemic areas within 1 month after returning to China, which would otherwise be classified as an indigenous case. For some special cases, such as cases reported from the Yunnan border region and cases without clear evidence to be identified as old or new infections, they will be discussed and classified by an expert group which was established by the National Health and Family Planning Commission (NHFPC) of the People’s Republic of China. Sometimes we also employed genotyping for case classification in the reference laboratory system, especially for the identification of non-vector-borne transmissions, such as infection by blood transfusion.

### Calculation of vulnerability

Vulnerability is calculated using the following equations:

1 $$ V={n}_a+{n}_I $$

2 $$ {D}_V=\frac{V}{N} $$

In these equations, *V* refers to vulnerability, *n*_*a*_ refers to the number of asymptomatic infections, *n*_*I*_ refers to reported imported cases, *D*_*V*_ refers to the density of vulnerability, and *N* refers to the number of inhabitants. In this study, *n*_*a*_ was estimated using the finger-prick blood samples that were collected during the cross-sectional survey. To avoid selection bias, *n*_*I*_ was estimated using the 4-year average for all imported cases from each village between January 2013 and September 2016.

### Model establishment

**Fig. 2:**
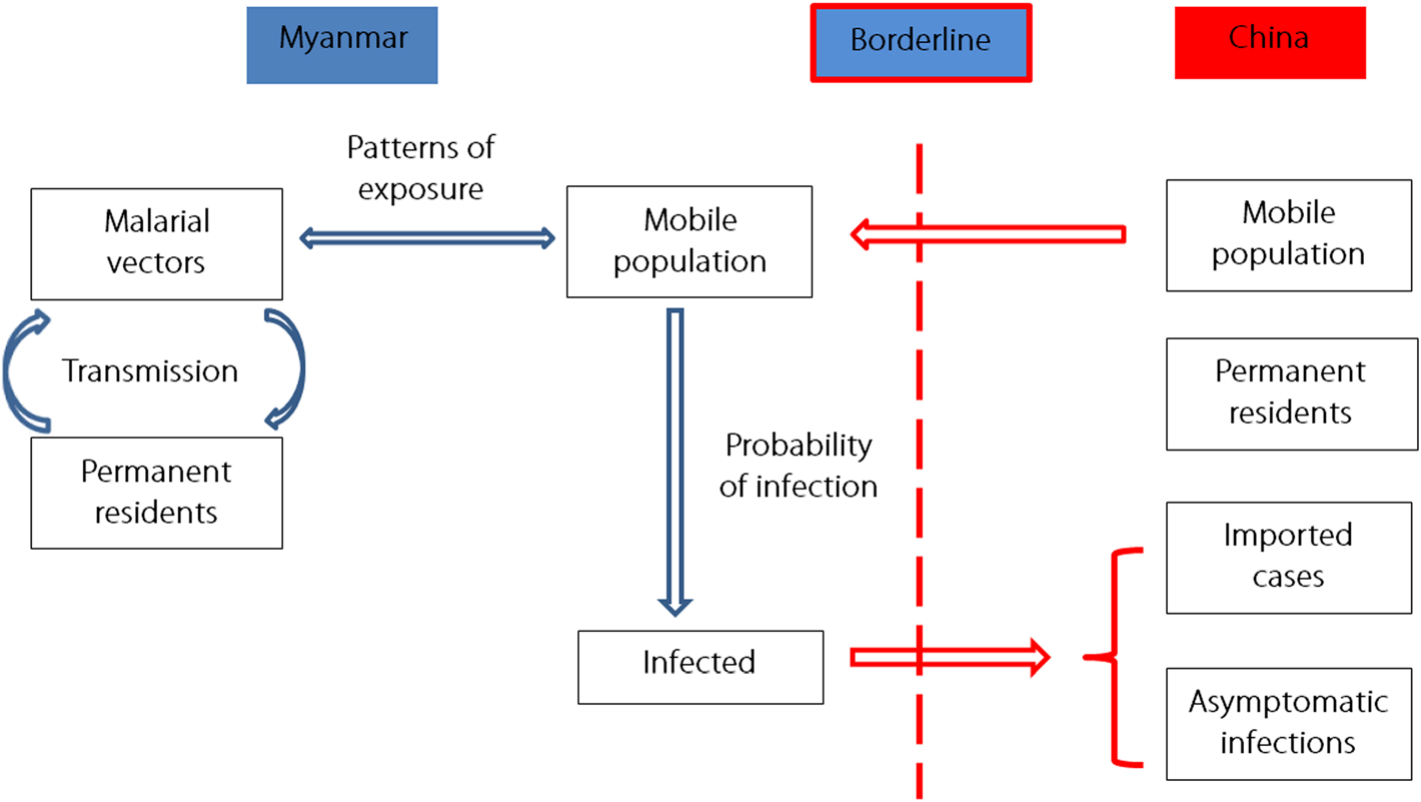
The simplified relationship between the mobile population and vulnerability to malaria at the China-Myanmar border

The stochastic simulation model (SSM) model was developed to simulate the relationship between population mobility and malaria vulnerability according the mechanism of malaria importation (Fig. 2). In the model, we assumed that malaria vulnerability could be affected by the proportion of the population that is mobile, the epidemic status of the areas for temporary immigration and emigration, the risks of exposure to malarial vectors, the efficacy of any protection measures, the duration of exposure, and the frequency of movement. The model used the following equations:

3 $$ D=\sum \limits_{j=1}^4\left({M}_j\times {T}_j\times {E}_j\times {p}_j\times q\times \left(1-e\right)\right) $$

4 $$ M=\frac{M_{im}+{M}_{em}}{N} $$

5 $$ T=f\times d $$

In the model, *D* is the density of imported cases (imported cases divided by total inhabitants), *M* is the mobile population proportion, *M*_*im*_ is the number of temporary immigrants, *M*_*em*_ is the number of temporary emigrants, *N* is the number of inhabitants, *T* is the total duration of exposure, *f* is the frequency of movement, *d* is the duration of each movement, *E* is the exposure risk, *p* is the probability of infection, and *e* is the efficacy of protection. We assumed that the probability of infection for mobile individuals who lived indoors at night should be multiplied by a protection coefficient (*q;* 0 < *q* < 1). The probability of infection (*p*) depends on the malaria situation in areas 1–4, which we defined as intense transmission (*j* = 1; ≥5 cases/1000 population), pre-elimination (*j* = 2; 1–4.9 cases/1000 population), elimination (*j* = 3; < 1 case/1000 population), and malaria-free (*j* = 4; no cases) based on the WHO World Malaria Report 2015. These areas included counties or global areas. For example, Myanmar may be included in area 1, and some villages in China where malaria is locally transmitted may be classified into area 3. To simulate the stochastic process for *p*, a random function [*f*(*x*) = random (0, 1)] was used with the condition that if *f*(*x*) > *p* × *q* × (1 - *e*), the individual would be considered infected. All other individuals were considered uninfected.

### Parameter estimation and simulation methods

Among the 9 parameters in the SSM model (*M*_*im*_, *M*_*em*_, *N*, *f*, *d*, *E*, *p*, *q*, and *e*), data regarding *M*_*im*_, *M*_*em*_, *N*, *f*, *d*, and *E* were obtained from the epidemiological survey. Data regarding the other 3 parameters (*p*, *q*, and *e*) were obtained using a model fitted with the vulnerability data from the selected villages. In the model fitting, the simulated *D* values were compared to the malaria vulnerability values (density of imported cases and asymptomatic infections) by calibrating each parameter until the chi-square test revealed no significant difference (*P* > 0.05).

To explore the relationship between malaria vulnerability and the proportion of the mobile population, the SSM model was simulated 1000 times using different *M* values (10%, 20%, …, 100%). This approach allowed us to obtain the simulated densities of imported infections based on different *M* values for each village.

Microsoft Office Excel 2010 (Microsoft Corp., Redmond, WA, USA) was used to run the SSM model. SPSS 13.0 (IBM Corp., Armonk, NY, USA) was used to perform the chi-square test and the Fisher’s exact test.

## Results

### Basic village information and the mobile populations

The five study villages were located in warm and rainy areas with variable terrain. The main crops produced by all five villages were banana and rice. The median number of permanent residents was 86 (range: 72–146), and 92.11% of the mobile population were temporary emigrants (Table 1). Among the immigrants, 66.67% moved from area 3 (the China-Myanmar border region in China), 26.67% moved from area 1 (Myanmar), and 6.67% moved from area 4 (an area with transmission interruption in China). In contrast, among the emigrants, 50.29% moved to area 1 (Myanmar or the Myanmar-Thailand border region), 25.71% moved to area 4 (an area with transmission interruption in China), and 24.00% moved to area 3 (the China-Myanmar border region in China). The differences in the geographical distributions of temporary emigrants were significant in the five villages, based on Pearson’s chi-square test (*χ*^2^ = 56.667, *P* < 0.001). The male mobile population was slightly larger than the female mobile population, although this difference was not significant in the five villages (*χ*^2^ = 0.225, *P* = 0.994). Most mobile individuals were 20–39-years-old, although no significant differences were observed in the age distributions for the five villages (Fisher’s exact test, *P* = 0.312).

### Malaria vulnerabilities in the five villages

During 2013–2016, 32 imported cases were reported in the five villages, with a 4-year average of one imported case per year (range: 0–5 cases). These cases predominantly involved *Plasmodium vivax* (93.75%) and generally involved male individuals (59.38%), although the cases were generally distributed equally among the seven age groups. The highest proportion of cases (50.00%) was detected in 2015. Fisher’s exact test revealed no significant differences among the five villages in their distributions of species (*P* = 1.000), sex (*P* = 0.651), age (*P* = 0.571), or temporal distribution (*P* = 0.233), which are all shown in Table 2. There was no significant difference between the reported cases in 2016 and the adjusted 4-year average number of cases (*χ*^2^ = 3.580, *P* = 0.466).

**Table 2:** Data and model for estimating vulnerability to malaria in the five selected villages

	Jing Po Zhai	Ka Ya He	Xin Cun	Zhuan Po Zhai	Hu Que Ba
Number of reported imported cases (2013–2016)	19	4	8	0	1
Species of malaria					
*P. vivax*	17	4	8	0	1
*P. falciparum*	2	0	0	0	0
Gender					
Male	10	2	6	0	1
Female	9	2	2	0	0
Age					
0–10	5	0	0	0	0
11–20	1	0	1	0	0
21–30	5	1	1	0	1
31–40	3	1	1	0	0
41–50	2	0	3	0	0
51–60	2	1	2	0	0
> 60	1	1	0	0	0
Year					
2013	0	0	0	0	0
2014	4	0	1	0	1
2015	9	4	3	0	0
2016	6	0	4	0	0
Four-year average reported imported cases (per year)	4.75	1.00	2.00	0.00	0.25
Number of blood samples collected	77	43	50	112	71
Number of tested asymptomatic infections	0	0	0	0	0
Density of vulnerability to malaria	0.03253	0.01163	0.02439	0.00000	0.00338
Simulated vulnerability to malaria	0.03248	0.01162	0.02438	0.00049	0.00338

No parasites were detected using PCR in the 353 blood samples from 2016. The median density of malaria vulnerability was 0.012 (range: 0.000–0.033) (Table 2).

### Parameter estimation and model fitting

The epidemiological survey revealed high proportions of reported mobile populations in each village (median: 32.56%, range: 28.38–71.95%). All reported temporary immigrants lived indoors at night with protection. Among the reported temporary emigrants, 78.29% lived indoors at night with protection, 1.14% lived indoors at night without protection, and 20.57% lived outdoors at night with protection. Most villages had similar patterns of exposure, except Xin Cun Village, where 78.26% of reported temporary emigrants lived outdoors at night with protection (Additional file 3). The average reported exposure time for the majority of the mobile population was < 5 months. The average reported frequency of movement was < 3 times in Jing Po Zhai, Ka Ya He, Zhuan Po Zhai, and Hu Que. Ba. However, relatively high values of both reported exposure time (11 months) and reported movement frequency (13 times) were observed in Xin Cun (Additional file 3).

The results of the model fitting revealed that the SSM model fit the reported data (*χ*^2^ = 0.487, *P* = 0.485). The median probabilities of infection were 0.011 in area 1 (range: 0.0048–0.1585) and 0.003 in area 3 (range: 0.0021–0.0038). The efficacy of protection was 20% and the protection coefficient (*p*) for living indoors at night was 0.95 (Additional file 3), which indicated that only 20% of protection against infection was associated with using protection measures (e.g., a screen door or window, repellent, normal bed nets, long-lasting insecticidal nets, or insecticide-treated nets), and that living indoors only provided 5% of protection against infection (vs. living outdoors at night).

### Simulated malaria vulnerabilities using different mobile population proportions

**Fig. 3:**
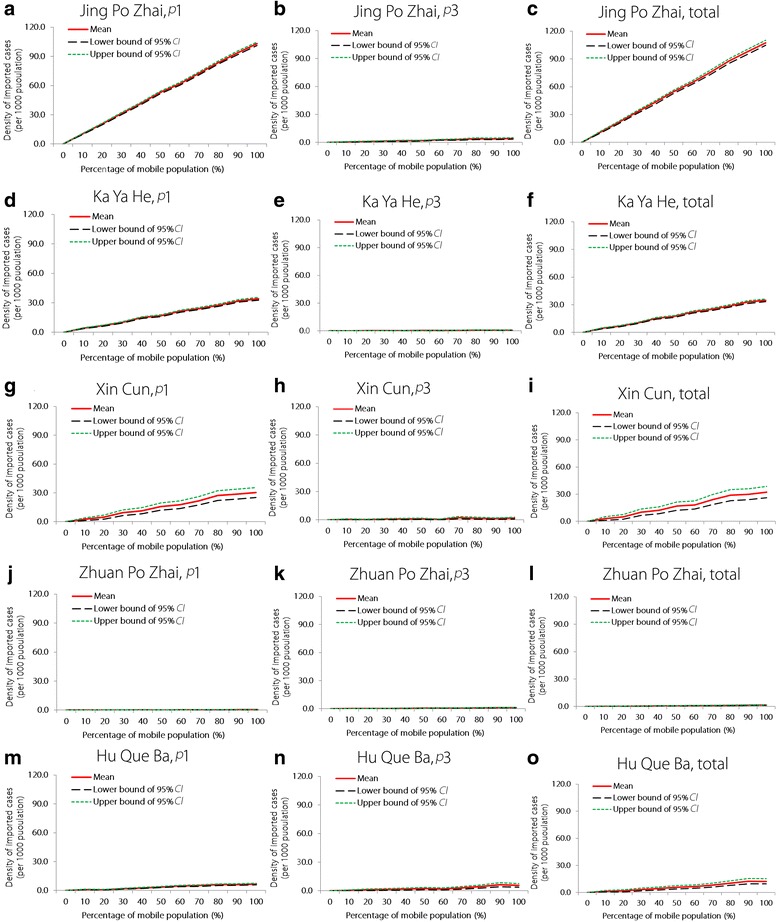
Simulated density of imported cases caused by varying proportions of mobility in five villages of Yingjiang county, China. Panels **a**-**o** represent the simulated density of imported cases in the five villages under the conditions of *p*_1_, * p*_3_, and the total probability, respectively. *p*_1_ indicates the probability of infection because of immigration or emigration from areas with the most intense transmission (≥5 cases per 1000 population). *p*_3_ indicates the probability of infection because of immigration or emigration from malaria elimination areas where the incidence is < 1 case per 1000 population. The total probability of infection is calculated based on immigration or emigration from all areas

Figure 3 shows that although the density of imported cases increased with the proportion of mobile individuals in a given population, each village had different vulnerability values. The highest simulated malaria vulnerabilities were observed in Jing Po Zhai, which was followed by Ka Ya He, Xin Cun, Hu Que. Ba, and Zhuan Po Zhai.

At medium (0.1 < *M* < 0.3) or high (0.3 ≤ *M* < 0.5) rates of migration from medium *p*_1_ areas (*p*_1_ < 0.1; Hu Que Ba and Zhuan Po Zhai), we found that malaria vulnerability increased slowly with the mobile population proportion. At exceedingly high (*M* ≥ 0.7) rates of migration from medium *p*_1_ areas (*p*_1_ < 0.1; Xin Cun), or high (0.3 ≤ *M* < 0.5) rates of migration from high *p*_1_ areas (0.1 ≤ *p*_1_ < 0.15; Ka Ya He), we found that malaria vulnerability increased with the mobile population proportion. At high (0.3 ≤ *M* < 0.5) rates of migration from exceedingly high *p*_1_ areas (*p*_1_ > 0.15; Jing Po Zhai), we found that malaria vulnerability increased dramatically with the mobile population proportion (Fig. 3).

**Fig. 4:**
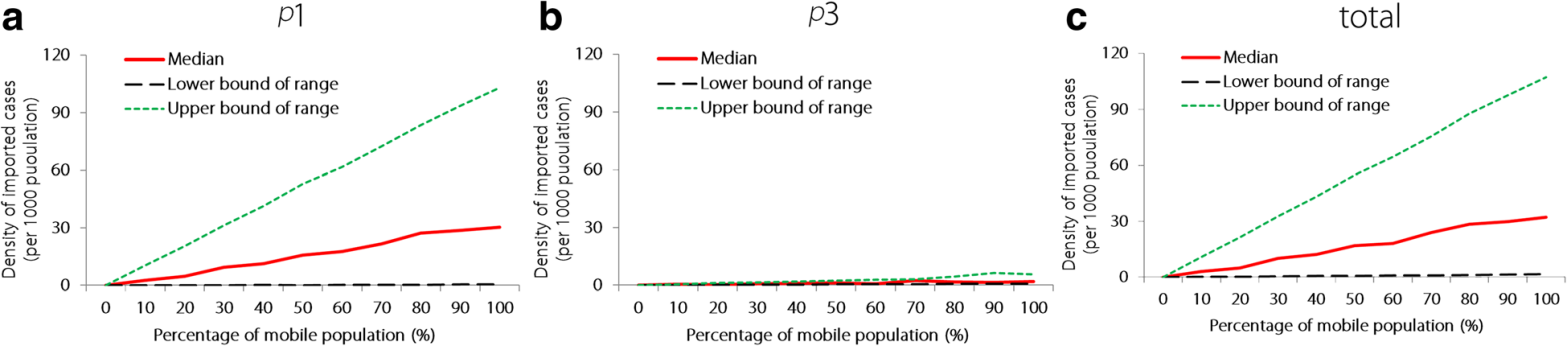
The median and range values for the simulated densities of imported cases caused by varying proportions of mobility. **a**, *p*_1_. **b**, *p*_3_. **c**, total. *p*_1_ indicates the probability of infection because of immigration or emigration from areas with the most intense transmission (≥5 cases per 1000 population). *p*_3_ indicates the probability of infection because of immigration or emigration from malaria elimination areas where the incidence is < 1 case per 1000 population. The total probability of infection is calculated based on immigration or emigration from all areas

Based on an average simulated *M* value of 10%, the imported case densities were 2.6/1000 population for area 1 (95% *CI*: 0.1–10.4/1000 population), 0.5/1000 population for area 3 (95% *CI*: 0.1–0.6/1000 population), and a total density of 3.2/1000 population (95% *CI*: 0.2–10.9/1000 population). The imported cases increased with an increasing proportion of mobile individuals in the study population (Fig. 4). Based on a simulated *M* value of 100%, the imported case densities increased to 30.5/1000 population for area 1 (95% *CI*: 0.4–103.1/1000 population), 1.9/1000 population for area 3 (95% *CI*: 0.7–5.7/1000 population), and a total density of 32.4/1000 population (95% *CI*: 1.6–107.4/1000 population).

## Discussion

Progress towards eliminating malaria in China has recently reduced the malaria transmission rate in Yunnan Province [25–27]. However, Yingjiang’s location in a warm and rainy area at the China-Myanmar border is associated with high malaria receptivity. Thus, malaria vulnerability has become a key factor for malaria re-establishment in this region. The present study revealed a high mobile population proportion in this county, with most mobile individuals temporarily moving to Myanmar. Fortunately, most mobile individuals lived indoors with protection, which may have reduced their risk of infection. However, our results indicate that living indoors only provided 5% of protection against infection (vs. living outdoors at night). The low efficacy of living indoors might be related to the several-hours period between sunset and actually going to sleep, as vectors could enter the house during this period; it is also possible that unclosed or leaky windows and doors contributed throughout the entire night. Nevertheless, the precise mechanism remains unclear, and additional research is needed to better understand why living indoors is associated with low efficacy. Our results also indicate that only 20% protection against infection was associated with using protection measures (e.g., a screen door or window, repellent, normal bed nets, long-lasting insecticidal nets, or insecticide-treated nets). The low efficacy of protection measures might be related to inappropriate use, and the period between sunset and going to sleep might be a critical time for infection. Countermeasures may be needed to decrease the infection of mobile people during this time. Another reason might be that people tend to over-report their use of bed nets and to underreport both their migration behaviors and living outdoors at night. This reporting bias from mobile populations might lead to an underestimation of the efficacy of living indoors and protection measures.

The high proportion of mobile individuals in the study population was associated with greater malaria vulnerability, which might increase the likelihood of malaria re-establishment in Yingjiang county. In addition, the model that was used for analysis fit the reported data, which indicates that the SSM model can be used to simulate the relationship between mobile population proportion and malaria vulnerability. Interestingly, we observed different infection probabilities among the mobile populations of the five villages, with the greatest probability observed in area 1. These differences might be related to the heterogeneous distribution of malaria transmission in Myanmar. Thus, the mobile populations of Jing Po Zhai, Ka Ya He, and Xin Cun might have moved to high transmission areas, while the mobile populations of Zhuan Po Zhai and Hu Que. Ba might have moved to low transmission areas in Myanmar.

Most mobile individuals lived indoors at night with protection, although the efficacy of protection and rate of sleeping indoors was low during their stays in Myanmar. Furthermore, we detected high values for the mobile population proportion and malaria vulnerability in the China-Myanmar border region. Moreover, the SSM model predicted a linear relationship between mobile population proportion and malaria vulnerability. Therefore, to reduce the risk of malaria re-establishment in the border regions of China, we recommend introducing mobile population-specific measures, such as health education to reduce malarial vector exposure and blood screening with ultrasensitive reverse transcription PCR to identify asymptomatic infections when mobile people return to China [28].

The present study is limited in that it only evaluated PCR-based data regarding asymptomatic infections from a single community-based, cross-sectional survey in 2016, and it is probable that asymptomatic infections were not detected during 2013–2015 when PCR was not used. Therefore, the regional vulnerability might be underestimated by using the 4-year average for imported cases, although we believe that this would only have a minor effect on our findings. It is important to match temporal behaviors with infection data from the same period, and the present cross-sectional study collected mobile population data from the previous year to ensure that it matched the imported case data from 2016. However, we found that no imported cases were reported in three villages (Table 2). Thus, to avoid selection bias by using only a single year for analysis, the data were adjusted using the 4-year average case numbers from each village during 2013–2016. After the adjustment, we found that SSM model fit the data and there was no significant difference between the reported cases in 2016 and the adjusted 4-year average number of cases, which indicates that the 4-year average is appropriate for use in the model.

The simulated results of our study are based on the assumptions of the SSM model. In this model, the independent variables included the proportion of mobile individuals in the study population, the epidemic status of the regions that have temporary immigration and emigration, the risk of exposure to malarial vectors, the efficacy of any protection measures, the duration of exposure, and the frequency of movement. However, there might be other independent variables or residual errors that should be considered in the model, and additional research is needed to more precisely explain the mechanism of malaria vulnerability.

Another limitation is the possibility of bias in linking *P. vivax* to migration patterns. For example, the high frequency of cross-border movement of mobile individuals and the long latent period of *P. vivax* infection [29] can make it difficult to determine when the *P. vivax* infection was acquired. Fortunately, China has developed a step-by-step protocol for case classification and a 5-level case confirmation network. The foundation of the network is comprised of each hospital and clinic that detects and reports malaria cases. Next, the county’s Center for Disease Control and Prevention (CDC) implements an epidemiological investigation to categorize the case as imported or indigenous. The municipal CDC then checks the information that was reported by the county CDC, and each case is finally verified by a provincial reference laboratory after being reported by the local public health institute. The final confirmation of each malaria case is approved by the NHFPC expert group. This process and the step-by-step protocol ensure that each malaria case is diagnosed and categorized correctly, and minimizes the likelihood of diagnostic bias in China.

## Conclusions

This community-based, cross-sectional study was performed to develop an SSM model that simulates mobile population dynamics and malaria vulnerability in the China-Myanmar border region. High population mobility was observed with different epidemiological characteristics and exposure patterns, which were associated with varying levels of vulnerability in the studied villages. Thus, the SSM model could be used as a tool to quantify the linear relationship between vulnerability and mobile populations, and it may be useful for assessing the risk of malaria re-establishment in China.

## Additional files

Additional file 1 Registration Form for Village Profile in Yingjiang County. (DOCX 27 kb)

Additional file 2 Questionnaire for Household Survey in Yingjiang County. (DOCX 29 kb)

Additional file 3 Parameter definitions and values for the five selected villages. (DOCX 42 kb)

## Abbreviations

CDC: Center for Disease Control and Prevention
IBM: Individual-based model
NHFPC: National Health and Family Planning Commission
PCR: Polymerase chain reaction
SSM: Stochastic simulation model
WHO: World Health Organization
