# Supplementary material for: Mobile population dynamics and malaria vulnerability: a modelling study in the China-Myanmar border region of Yunnan Province, China
Source: Infect Dis Poverty. 2018 Apr 29;7:36. doi: 10.1186/s40249-018-0423-6 (PMC5924679; doi:10.1186/s40249-018-0423-6)
Supplement: Supplementary file 1 — Registration Form for Village Profile in Yingjiang County. (DOCX 27 kb) [file 40249_2018_423_MOESM1_ESM.docx]

**Additional file 1. Registration Form for Village Profile in Yingjiang County**

| **I1. Investigator** | | | | | Name: | | | | | | | | | | |  |
| --- | --- | --- | --- | --- | --- | --- | --- | --- | --- | --- | --- | --- | --- | --- | --- | --- |
| **I2. Investigating date** | | | | | □□□□-□□-□□ (YYYY-MM-DD) | | | | | | | | | | |  |
| **I3. Basic information** | **I3.1 Township** | | | | Name: Code: □□ | | | | | | | | | | |  |
|  | **I3.2 Administrative village** | | | | Name: Code: □□ | | | | | | | | | | |  |
|  | **I3.3 Natural village** | | | | Name: Code: □□ | | | | | | | | | | |  |
|  | **I3.4 GPS coordinates** | | | | N□□.□□□□□ E□□.□□□□□ | | | | | | | | | | |  |
|  | **I3.5** **Elevation** | | | | □□□□m | | | | | | | | | | |  |
|  | **I3.6 Landform** | | | | 1=Plain 2=Mountain 3=River network 4=Hill  5=Basin 6=Valley 7=Others (Please specify) | | | | | | | | □ | | |  |
|  | **I3.7 Area** | | | | □□□□km^2^ | | | | | | | | | | |  |
|  | **I3.8 Cultivated area** | | | | □□□□mu, including paddy field □□□□mu | | | | | | | | | | |  |
|  | **I3.9 Main crops** | | | | 1=Paddy rice 2=Maize 3=Sugarcane 4=Banana 5=Others | | | | | | | | □ | | |  |
|  | **I3.10** **Annual average temperature** | | | | □□.□□℃ | | | | | | | | | | |  |
|  | **I3.11** **Annual rainfall** | | | | □□□□mm | | | | | | | | | | |  |
|  | **I3.12** **Annual average relative humidity** | | | | □□.□□％ | | | | | | | | | | |  |
|  | **I3.13 No. of household** | | | | □□ | | | | | | | | | | |  |
|  | **I3.14** **Registered population** | | | | □□□ | | | | | | | | | | |  |
|  | **I3.15 Resident population** | | | | □□□ | | | | | | | | | | |  |
|  | **I3.16 Mobile population** | | | | □□□; immigrant:□□□, emigrant:□□□ | | | | | | | | | | |  |
|  |  | | | |  | | | | | | | | | | |  |
| **I4. Malaria cases in 2013-2016** | **I4.1 Year**  YYYY | **I4.2 GPS coordinates**  N□□.□□□□□  E□□.□□□□□ | **I4.3 Head of household** | **I4.4 Patient name** | | **I4.5 Gender**  1=Male 2=Female | **I4.6 Birth date**  YYYY-MM-DD | **I4.7 Diagnosis result**  1=Clinical 2=Pv 3=Pf  4=Po 5=Pm 6=Mixed | **I4.8 Infected place**  1=Indigenous 2=Other county  3=Other Province 4=Abroad | **I4.9 Onset date**  YYYY-MM-DD | **I4.10 Diagnosis date**  YYYY-MM-DD | **I4.11 Reporting date**  YYYY-MM-DD | | **I4.12 Case investigating date**  YYYY-MM-DD | **I4.13 Foci disposal date**  YYYY-MM-DD | |
|  |  |  |  |  | |  |  |  |  |  |  |  | |  |  | |
|  |  |  |  |  | |  |  |  |  |  |  |  | |  |  | |
|  |  |  |  |  | |  |  |  |  |  |  |  | |  |  | |
|  |  |  |  |  | |  |  |  |  |  |  |  | |  |  | |
|  |  |  |  |  | |  |  |  |  |  |  |  | |  |  | |
|  |  |  |  |  | |  |  |  |  |  |  |  | |  |  | |
|  |  |  |  |  | |  |  |  |  |  |  |  | |  |  | |
|  |  |  |  |  | |  |  |  |  |  |  |  | |  |  | |
|  |  |  |  |  | |  |  |  |  |  |  |  | |  |  | |
