# Supplementary material for: Mobile population dynamics and malaria vulnerability: a modelling study in the China-Myanmar border region of Yunnan Province, China
Source: Infect Dis Poverty. 2018 Apr 29;7:36. doi: 10.1186/s40249-018-0423-6 (PMC5924679; doi:10.1186/s40249-018-0423-6)
Supplement: Supplementary file 2 — Questionnaire for Household Survey in Yingjiang County. (DOCX 29 kb) [file 40249_2018_423_MOESM2_ESM.docx]

**Additional file 2. Questionnaire for Household Survey in Yingjiang County**

| **I1. Investigator** | Blood sample collector: Note-taker: | | | | | |
| --- | --- | --- | --- | --- | --- | --- |
| **I2. Investigating date** | □□□□-□□-□□（YYYY-MM-DD） | | | | | |
| **I3. Township** | Name: Code: □□ | | | | | |
| **I4. Administrative village** | Name: Code: □□ | | | | | |
| **I5. Natural village** | Name: Code: □□ | | | | | |
| **I6. Household** | Head: Code: □□  GPS Coordinates: N□□.□□□□□ E□□.□□□□□  Population: □□ | | | | | |
| **I7. Name of family members** | |  |  |  |  |  |
| **I8. Code** | | 01 | 02 | 03 | 04 | 05 |
| **I9. Gender**  1=Male 2=Female | |  |  |  |  |  |
| **I10. Age** | | □□Years  □□Months | □□Years  □□Months | □□Years  □□Months | □□Years  □□Months | □□Years  □□Months |
| **I11. Nationality** | |  |  |  |  |  |
| **I12. Occupation**  1=Preprimary children  2=Student 3=Farmer  4=Labor 5=Business man  6=Teacher 7=Civil servant  8=others (Please specify) | |  |  |  |  |  |
| **I13. Education**  1=Illiteracy 2=Preprimary  3=Primary school  4=Junior high school  5=Senior high school  6=Technical secondary school  7=Junior college and above | |  |  |  |  |  |
| **I14. Is temporary visitor?**  1=Yes 2=No | |  |  |  |  |  |
| **I15. Where is the visitor from?** | | County:  Province:  County:  Township  Village: | County:  Province:  County:  Township  Village: | County:  Province:  County:  Township  Village: | County:  Province:  County:  Township  Village: | County:  Province:  County:  Township  Village: |
| **I16. How often stayed?** | | □□Times | □□Times | □□Times | □□Times | □□Times |
| **I17. How long stayed?** | | □□Months | □□Months | □□Months | □□Months | □□Months |
| **I18. How long to stay?** | | □□Months | □□Months | □□Months | □□Months | □□Months |
| **I19. Where travelled?** | | County:  Province:  County:  Township  Village: | County:  Province:  County:  Township  Village: | County:  Province:  County:  Township  Village: | County:  Province:  County:  Township  Village: | County:  Province:  County:  Township  Village: |
| **I20. How often travelled?** | | □□Times | □□Times | □□Times | □□Times | □□Times |
| **I21. How long travelled?** | | □□Months | □□Months | □□Months | □□Months | □□Months |
| **I22. Have heard of Malaria?**  1=Yes 2=No | |  |  |  |  |  |
| **I23. How malaria transmitted?**  1=Contact 2=Food  3=Mosquito biting  4=Drinking water 5=Air  6=Others (Please specify)  7=Don’t know | |  |  |  |  |  |
| **I24. What to do when getting malaria?**  1=See doctors  2=Medication from drugstores  3=Do not see doctors or take medicines  4=Others (Please specify)  5=Don’t know | |  |  |  |  |  |
| **I25. How to prevent malaria?**  1=No contact with patients  2=No insanitary eating or drinking  3=Keep from mosquito biting  4=Wear a mask  5=Others (Please specify)  6=Don’t know | |  |  |  |  |  |
| **I26. Outdoor sleeping habit**  1=Yes 2=No | |  |  |  |  |  |
| **I27. Using screen door**  1=Yes 2=No | |  |  |  |  |  |
| **I28. Using screen window**  1=Yes 2=No | |  |  |  |  |  |
| **I29. Using bed-net**  1=LLIN 2=ITN  3=Conventional bed-net  4=No | |  |  |  |  |  |
| **I30. Using repellents**  1=Yes 2=No | |  |  |  |  |  |
| **I31. Traveling out of home and stayed overnight within the past month**  1=Yes 2=No | |  |  |  |  |  |
| **I32. Where travelled?** | | County:  Province:  County:  Township  Village: | County:  Province:  County:  Township  Village: | County:  Province:  County:  Township  Village: | County:  Province:  County:  Township  Village: | County:  Province:  County:  Township  Village: |
| **I33. Protections when travelling**  1=Using bed-net  2=Using repellent  3=Taking chemoprophylaxis  4=Wear long clothes  5=No precautions  6=Others (Please specify) | |  |  |  |  |  |
| **I34. Having fever within the past 2 weeks**  1=Yes 2=No | |  |  |  |  |  |
| **I35. Where got diagnosed?**  1=Self diagnosis  2=Private doctors  3=Drugstore  4=Village clinic  5=Township hospital  6=County hospital  7=County CDC  8=Others (Please specify) | |  |  |  |  |  |
| **I36. Blood sampling**  1=Prink finger blood sample  2=Not sampled | |  |  |  |  |  |
