# Supplementary material for: Mobile population dynamics and malaria vulnerability: a modelling study in the China-Myanmar border region of Yunnan Province, China
Source: Infect Dis Poverty. 2018 Apr 29;7:36. doi: 10.1186/s40249-018-0423-6 (PMC5924679; doi:10.1186/s40249-018-0423-6)
Supplement: Supplementary file 3 — Parameter definitions and values for the five selected villages. (DOCX 42 kb) [file 40249_2018_423_MOESM3_ESM.docx]

**Additional file 3. Parameter definitions and values for the five selected villages.**

| Parameter | Description | Value | | | | | Method |
| --- | --- | --- | --- | --- | --- | --- | --- |
|  |  | Jing Po Zhai | Ka Ya He | Xin Cun | Zhuan Po Zhai | Hu Que Ba |  |
| *M* | Density of mobile population | 0.3151 | 0.3256 | 0.7195 | 0.3364 | 0.2838 | Field epidemiological survey |
| *M_im_* | Immigrant | 0.0274 | 0.0233 | 0.0000 | 0.0561 | 0.0405 | Field epidemiological survey |
| *M_em_* | Emigrant | 0.2877 | 0.3023 | 0.7195 | 0.2804 | 0.2432 | Field epidemiological survey |
| *E_I_* | Pattern of exposure (Immigrant) |  |  |  |  |  | Field epidemiological survey |
|  | LON with protection | 0 | 0 | 0 | 0 | 0 |  |
|  | LON without protection | 0 | 0 | 0 | 0 | 0 |  |
|  | LIN with protection | 4 | 2 | 0 | 6 | 3 |  |
|  | LIN without protection | 0 | 0 | 0 | 0 | 0 |  |
| *E_I_*_1_ | Pattern of exposure (area 1) |  |  |  |  |  | Field epidemiological survey |
|  | LON with protection | 0 | 0 | 0 | 0 | 0 |  |
|  | LON without protection | 0 | 0 | 0 | 0 | 0 |  |
|  | LIN with protection | 4 | 0 | 0 | 0 | 0 |  |
|  | LIN without protection | 0 | 0 | 0 | 0 | 0 |  |
| *E_I_*_2_ | Pattern of exposure (area 2) |  |  |  |  |  | Field epidemiological survey |
|  | LON with protection | 0 | 0 | 0 | 0 | 0 |  |
|  | LON without protection | 0 | 0 | 0 | 0 | 0 |  |
|  | LIN with protection | 0 | 0 | 0 | 0 | 0 |  |
|  | LIN without protection | 0 | 0 | 0 | 0 | 0 |  |
| *E_I_*_3_ | Pattern of exposure (area 3) |  |  |  |  |  | Field epidemiological survey |
|  | LON with protection | 0 | 0 | 0 | 0 | 0 |  |
|  | LON without protection | 0 | 0 | 0 | 0 | 0 |  |
|  | LIN with protection | 0 | 2 | 0 | 6 | 2 |  |
|  | LIN without protection | 0 | 0 | 0 | 0 | 0 |  |
| *E_I_*_4_ | Pattern of exposure (area 4) |  |  |  |  |  | Field epidemiological survey |
|  | LON with protection | 0 | 0 | 0 | 0 | 0 |  |
|  | LON without protection | 0 | 0 | 0 | 0 | 0 |  |
|  | LIN with protection | 0 | 0 | 0 | 0 | 1 |  |
|  | LIN without protection | 0 | 0 | 0 | 0 | 0 |  |
| *E_E_* | Pattern of exposure (Emigrant) |  |  |  |  |  | Field epidemiological survey |
|  | LON with protection | 0 | 0 | 36 | 0 | 0 |  |
|  | LON without protection | 0 | 0 | 0 | 0 | 0 |  |
|  | LIN with protection | 41 | 26 | 22 | 30 | 18 |  |
|  | LIN without protection | 1 | 0 | 1 | 0 | 0 |  |
| *E_E_*_1_ | Pattern of exposure (area 1) |  |  |  |  |  | Field epidemiological survey |
|  | LON with protection | 0 | 0 | 36 | 0 | 0 |  |
|  | LON without protection | 0 | 0 | 0 | 0 | 0 |  |
|  | LIN with protection | 23 | 8 | 10 | 3 | 8 |  |
|  | LIN without protection | 0 | 0 | 0 | 0 | 0 |  |
| *E_E_*_2_ | Pattern of exposure (area 2) |  |  |  |  |  | Field epidemiological survey |
|  | LON with protection | 0 | 0 | 0 | 0 | 0 |  |
|  | LON without protection | 0 | 0 | 0 | 0 | 0 |  |
|  | LIN with protection | 0 | 0 | 0 | 0 | 0 |  |
|  | LIN without protection | 0 | 0 | 0 | 0 | 0 |  |
| *E_E_*_3_ | Pattern of exposure (area 3) |  |  |  |  |  | Field epidemiological survey |
|  | LON with protection | 0 | 0 | 0 | 0 | 0 |  |
|  | LON without protection | 0 | 0 | 0 | 0 | 0 |  |
|  | LIN with protection | 12 | 6 | 6 | 17 | 1 |  |
|  | LIN without protection | 0 | 0 | 0 | 0 | 0 |  |
| *E_E_*_4_ | Pattern of exposure (area 4) |  |  |  |  |  | Field epidemiological survey |
|  | LON with protection | 0 | 0 | 0 | 0 | 0 |  |
|  | LON without protection | 0 | 0 | 0 | 0 | 0 |  |
|  | LIN with protection | 6 | 12 | 6 | 10 | 9 |  |
|  | LIN without protection | 1 | 0 | 1 | 0 | 0 |  |
| *T* | Average time of exposure (month) | 4.0 | 1.6 | 11.0 | 2.1 | 4.6 | Field epidemiological survey |
| *f* | Avg. frequency | 2.2 | 2.5 | 12.9 | 2.4 | 2.0 | Field epidemiological survey |
| *d* | Avg. duration per movement | 1.9 | 0.7 | 0.9 | 0.9 | 2.3 | Field epidemiological survey |
| *T*_1_ | Average time of exposure (area 1) | 1.4 | 1.3 | 10.6 | 1.0 | 2.2 | Field epidemiological survey |
| *f*_1_ | Avg. frequency | 2.8 | 3.3 | 15.6 | 1.0 | 3.1 | Field epidemiological survey |
| *d*_1_ | Avg. duration per movement | 0.5 | 0.4 | 0.7 | 1.0 | 0.7 | Field epidemiological survey |
| *T*_2_ | Average time of exposure (area 2) | 0.0 | 0.0 | 0.0 | 0.0 | 0.0 | Field epidemiological survey |
| *f*_2_ | Avg. frequency | 0.0 | 0.0 | 0.0 | 0.0 | 0.0 | Field epidemiological survey |
| *d*_2_ | Avg. duration per movement | 0.0 | 0.0 | 0.0 | 0.0 | 0.0 | Field epidemiological survey |
| *T*_3_ | Average time of exposure (area 3) | 6.9 | 1.0 | 12.0 | 1.1 | 12.0 | Field epidemiological survey |
| *f*_3_ | Avg. frequency | 1.0 | 1.3 | 5.7 | 3.2 | 1.0 | Field epidemiological survey |
| *d*_3_ | Avg. duration per movement | 6.9 | 0.8 | 2.1 | 0.3 | 12.0 | Field epidemiological survey |
| *T*_4_ | Average time of exposure (area 4) | 7.6 | 2.2 | 12.0 | 4.2 | 5.9 | Field epidemiological survey |
| *f*_4_ | Avg. frequency | 2.0 | 2.5 | 1.7 | 1.4 | 1.1 | Field epidemiological survey |
| *d*_4_ | Avg. duration per movement | 3.8 | 0.9 | 7.0 | 3.0 | 5.3 | Field epidemiological survey |
| *p* | Probability of infection |  |  |  |  |  | Model fitting |
| *p*_1_ | Probability in area 1 | 0.1585 | 0.1285 | 0.0048 | 0.0058 | 0.0110 |  |
| *p*_2_ | Probability in area 2 | NA | NA | NA | NA | NA |  |
| *p*_3_ | Probability in area 3 | 0.0030 | 0.0037 | 0.0026 | 0.0021 | 0.0038 |  |
| *p*_4_ | Probability in area 4 | 0 | 0 | 0 | 0 | 0 |  |
| *e* | Efficacy of protection | 0.20 | 0.20 | 0.20 | 0.20 | 0.20 | Model fitting |
| *q* | Protection coefficient of living indoor compared to living outdoor at night | 0.95 | 0.95 | 0.95 | 0.95 | 0.95 | Model fitting |

LON, living outdoor at night; LIN, living indoor at night; Avg., average; NA, not available.
